# Physicochemical and Active Properties of Gelatine-Based Composite Gels Loaded with Lysozyme and Green Tea Polyphenols

**DOI:** 10.17113/ftb.59.03.21.7029

**Published:** 2021-09

**Authors:** Derya Boyacı, Pelin Barış Kavur, Sukru Gulec, Ahmet Yemenicioğlu

**Affiliations:** 1 Department of Food Engineering, Izmir Institute of Technology, 35430 Gulbahce Koyu, Urla, Izmir, Turkey; 2 School of Engineering, University of Lincoln, LN6 7TS Brayford Pool, Lincoln, United Kingdom; 3 Molecular Nutrition and Human Physiology Laboratory, Faculty of Engineering, Izmir Institute of Technology, 35430 Gulbahce Koyu, Urla, Izmir, Turkey

**Keywords:** gelatine gel, candelilla wax, rice starch, lysozyme, composite gel, green tea extract

## Abstract

**Research background:**

The use of gel-based systems as a novel method for the delivery of natural antimicrobial, antioxidant and bioactive compounds is a developing innovative solution for the food industry. This research aims to develop multifunctional active edible gels based on gelatine and its composites with improved mechanical properties.

**Experimental approach:**

Antilisterial and bioactive composite gels showing different physical and active properties from classical gelatine gel were developed by loading lysozyme and green tea extract into gelatine/starch and gelatine/wax composite gels. Mechanical properties, swelling profiles, colour, release profiles, and antimicrobial and bioactive properties of the gels were characterised.

**Results and conclusions:**

Gelatine/wax gels showed 1.3- to 2.1-fold higher firmness and cutting strength than gelatine and gelatine/starch composite gels that had similar firmness and cutting strengths. Work to shear of both composite gels was 1.4- to 1.9-fold higher than that of gelatine gel. The gelatine/starch gel showed the highest water absorption capacity. Green tea extract reduced soluble lysozyme in all gels, but composite gels contained higher amount of soluble lysozyme than gelatine gel. All the gels with lysozyme inhibited *Listeria innocua* growth in the broth media, while green tea extract showed antilisterial activity only in gelatine/wax gels. Gels with green tea extract showed antioxidant, antidiabetic (α-glucosidase and α-amylase inhibition), antihypertensive (angiotensin-converting enzyme inhibition) and antiproliferative activities (on Caco-2 human colon carcinoma cells). However, gelatine and gelatine/wax gels showed the highest antioxidant and antidiabetic activity. The gelatine/wax gels prevented phenolic browning, while green tea extract in other gels showed moderate or extensive browning.

**Novelty and scientific contribution:**

This work clearly showed the possibility of improving mechanical properties and modifying water absorption and controlled release profiles of gelatine gels using gelatine/starch and gelatine/wax composites. The novel composite gels reduced browning of incorporated polyphenols and showed antilisterial and bioactive properties.

## INTRODUCTION

The use of gel-based systems for delivery of natural antimicrobials, antioxidants and bioactive compounds has gained an increased interest since such systems could find innovative applications in food, biomedical and pharmaceutical sectors (*1*-*4*). Gelatine is the most indispensable animal-based food hydrocolloid that has been extensively used not only for its unique gelation capacity but also for its elasticity, texture, taste and nutritive value (*5*). However, the low mechanical stability of gelatine-based food and materials, as well as incompatibility of gelatine with some bioactive polyphenols (*e.g.* it darkens in contact with polyphenols), interferes with its innovative food applications to obtain novel functional gel-based food and bioactive materials (*e.g.* film, pad, coating or filling materials) suitable for delivery of active compounds (*1*, *4*, *6*). Therefore, different efforts have been made to develop more mechanically stable composites of gelatine with proteins (*e.g.* soy protein isolate), polysaccharides (*e.g.* starch, alginate, xanthan, carboxymethyl cellulose, gellan, sucrose and inulin) and waxes that could be employed as antimicrobial filling, coating or pad materials (*1*, *4*, *7*-*11*). Gelatine is also extensively used to obtain numerous gel-based foods such as fruit jellies prepared with fresh fruits, toppings for pâté, and aspic food obtained by glazing, coating or embedding ready-to-eat animal source foods such as meat, chicken, pork, fish and eggs with gelatine. Microbial safety of such gel-based products is extremely important since they are cold-stored for some time, and this provides a perfect medium for the growth of pathogenic bacteria like *Listeria monocytogenes* that causes deadly infections in pregnant women, elderly and immunosuppressed people (*12*, *13*). The most severe effects of this bacterium were observed in the past in pâté and pork tongue in aspic with large-scale invasive listeriosis outbreaks in England (1988) and France (1992), respectively (*14*).

The main objective of the current study is to develop antilisterial and bioactive gelatine-based composite gels having different physical and active properties from those of classical gelatine gels. Such composite gels could serve to develop alternative safer and healthier gel-based foods and edible active filling, coating, glazing or pad materials. The composite gels of gelatine with hydrophilic rice starch and hydrophobic candelilla wax were prepared mainly to improve the poor mechanical properties of gelatine gel, compatibility of gelatine with polyphenols, and to modify its functional, visual and water absorption properties. The lysozyme and green tea extract were used as model natural compounds to obtain antimicrobial and bioactive properties of the gels. Both lysozyme and green tea extract are proven antilisterial agents (*15*-*19*). Antilisterial capacities of the gels were characterised using *L. innocua* as a surrogate for *L. monocytogenes* which might pose serious risks for the laboratory staff if contamination occurs. Moreover, green tea extract is a perfect source of catechins (*e.g.* catechin, epicatechin, epicatechin gallate and epigallocatechin gallate) that have been increasingly used in foods due to their well-characterised molecular structure, bioavailability and *in vivo* and *in vitro* health benefits (*20*, *21*). To evaluate their potential health benefits, the developed gels were characterised for well-known bioactive properties of green tea extract such as antioxidant, antidiabetic, antihypertensive and antiproliferative activities. This work is original in that it is the first study that focused not only on improving antimicrobial and bioactive properties of classical gelatine gel, but also on developing its weak physical and mechanical properties, as well as incompatibility with polyphenols that limit its various applications.

## MATERIALS AND METHODS

### Materials

Bovine skin gelatine (type B, 225 g Bloom gel strength), rice starch, candelilla wax, lysozyme from hen egg white (activity by producer: ≥40 000 U/mg), 2,2′-azino-bis(3-ethylbenzothiazoline-6-sulfonic acid) diammonium salt (ABTS), N-[3-(2-furyl)acryloyl]-l-phenylalanyl-glyciyl-glycine (FAPGG), 2,2-azobis(2-amidinopropane) dihydrochloride (AAPH), angiotensin-converting enzyme from rabbit lung, α-amylase from human saliva, and rat intestine acetone powder were obtained from Sigma-Aldrich, Merck (St. Louis, MO, USA). Green tea extract (100%) with a minimum of 22% total polyphenol content was obtained from Wild Flavours and Specialty Ingredients (Rudolf Wild GmbH & Co. KG, Eppelheim, Germany). Caco-2 cells used in cytotoxicity assay were obtained from ATCC, Manassas, VA, USA. *Listeria innocua* (NRRL B-33314) used in antimicrobial tests was obtained from the United States Department of Agriculture, Microbial Genomics and Bioprocessing Research Unit (Peoria, IL, USA).

### Preparation of gelatine and gelatine-based composite gels

Gel solutions were prepared by dissolving gelatine (15%, by mass) in distilled water (55 °C) by stirring at 500 rpm. Rice starch or candelilla wax (7.5%, by mass) was added into the gelatine solution to produce gelatine/starch and gelatine/wax composite gels, respectively. The gelatine and gelatine/starch solutions were homogenized at 10 000 rpm for 1 min using a homogenizer (Silent Crusher M with rotor *d*=6.6 mm tip; Heidolph Instruments, Schwabach, Germany). All gel-forming solutions were heated in a water bath at 85 °C for 30 min. Gelatine/wax mixture was homogenized at 10 000 rpm for 1 min to distribute melted wax within the gelatine solution. After cooling, green tea extract and lysozyme were added to the gel solutions at amount of 1% (by mass). All mixtures were further homogenized at 10 000 rpm for 3 min to distribute active agents homogenously. The gel-forming solutions were then poured into moulds and incubated for 20 h at 4 °C to achieve complete gelation.

### Mechanical properties (shear test)

A shear test was performed to determine the mechanical properties of gels using a TA.XT plus texture analyser (Stable Micro Systems Ltd., Godalming, UK) equipped with a blade set (HDP/BS) with knife/guillotine probe (crosshead speed: 200 mm/min, cell load: 5 kg). Test conditions used by Muñoz *et al.* (*22*) were applied with slight modifications. The gel samples were prepared by pouring 50 g of gel-forming solution into cubic moulds (5 cm^3^) and incubating for 20 h at 4 °C. The gel samples were cut into 2.5 cm^3^ portions and brought to 4 °C before testing. The gels were sheared through the centre, and their cutting strength (N/mm), maximum shear force (firmness) (N), and area under the curve (work to shear) (N·s) were determined from the force *vs* time curve. The experiments of each sample were replicated twice with four repetitions.

### Water-binding capacity

Water-binding capacity (WBC) of the gels was determined according to the gravimetric method (*23*). For analysis, 10 g of gels cast into plastic Petri dishes (*d*=6.6 cm, *δ*=0.5 cm) were weighed (*m*_1_) and placed into 100 mL of distilled water, and they were incubated at 4 °C under shaking at 80×*g* for 15 days. The gels were taken out of the water every 24 h and weighed (*m*_2_). Each gel was weighed in triplicate. WBC was calculated using the following equation:

WBC=(*m*_2_–*m*_1_)/*m*_1_ /1/

where *m*_1_ is the initial mass and *m*_2_ is the mass of gel in g at the equilibrium.

### Colour of gels

Colour of the gels was determined using a digital colourimeter (chromometer type CR-400; Konica Minolta, Tokyo, Japan) standardized with a white plate (Y=93.80, X=0.3159, y=0.3322). For analysis, 50 g of cubic gel samples were used. Results were expressed with CIE (Commission International de l’Eclairage): L* (0=dark, 100=light), a* (-*a*=greenness, +*a*=redness, 0=neutral) and b* (-*b*=blueness; +*b*=yellowness, 0=neutral).

### Release profile analysis

To determine the release profiles of lysozyme and green tea extract polyphenols, discs of gels (*m*=~10 g, *δ*=0.5 cm, *d*=6.6 cm) were placed into Erlenmeyer flasks that contained 100 mL of distilled water at 4 °C. The flasks were then shaken at 80 rpm, and the samples (0.1 mL) collected at different time intervals were tested for lysozyme activity or total phenolic content until reaching the equilibrium. The lysozyme activity was measured spectrophotometrically (model 2450; Shimadzu, Tokyo, Japan) at 660 nm using *Micrococcus lysodeicticus* as a substrate as described by Boyacı *et al*. (*1*). The released total phenolic content was measured spectrophotometrically (at 795 nm) according to the standard Folin-Ciocalteu method (*24*). Catechin was used as a standard for the determination of phenolic compounds. The measurements were performed as two replicates and three parallels. All calculations were corrected by considering the activity removed by the collected aliquots during sampling. The total lysozyme activity and total phenolic content released from each gel corresponded to maximum units (U) and maximum phenolic content (as mg catechin) released per g of gels at the equilibrium, respectively. The release curves were formed by plotting the calculated released lysozyme activities (U/g) or phenolic contents (mg/g) from gels *vs* time (h). The initial release rates of lysozyme and green tea extract phenolics were determined from the slope of the initial linear portion of release curves in U/(g·h) and mg/(g·h), respectively. The recovery of lysozyme and green tea extract from the gels was determined from the formula given in the following equation:

Lysozyme or polyphenol recovery=(Total lysozyme activity or total phenolic content released/Total lysozyme activity or total polyphenol added into gels)∙100 /2/

According to the methods described in this study, adding 1% lysozyme and green tea extract in gels resulted in an initial lysozyme activity of 744 194 U and initial mass fraction of 3.29 mg total polyphenols expressed as catechin equivalents per g of gels.

### Antimicrobial activity in broth media

The antimicrobial activity of gel discs (*m*=~10 g, *δ*=0.5 cm, *d*=6.6 cm) was tested against *Listeria innocua* during 48-hour incubation by the shake flask method (at 80 rpm) in 100 mL of nutrient broth at 4 °C (*1*). Microbiological counts were expressed as log CFU/mL, and the mean values and standard errors were calculated. At least three plates were enumerated for the calculations.

### Bioactive properties

The bioactive properties of the gels were expressed based on the total phenolic content of the released green tea extract from the gels during the release profile analysis. Thus, the release media that reached equilibrium of the polyphenol content were used directly to determine the antioxidant, antihypertensive and antidiabetic activities of the green tea extract released from the gels. For antiproliferative activity tests, the release media equilibrated for the phenolic compounds were lyophilized using a freeze drier (Freezone 6L; Labconco, Kansas City, MO, USA). The stock solutions used in antiproliferative activity were prepared freshly by dissolving lyophilized samples in ultrapure water.

#### Antioxidant activity

Trolox equivalent antioxidant capacity (TEAC) of active agents released form the gels was measured spectrophotometrically (model 2450; Shimadzu, Tokyo, Japan) using ABTS radical solution (*25*). Oxygen radical absorbance capacity (ORAC) was determined using AAPH solution as reaction initiator and the fluorescence of the reaction mixture was monitored using a microplate reader (Varioskan Flash; Thermo Fisher Scientific, Waltham, MA, USA) (*26*). The iron-chelating capacity (ICC) of active compounds released from the gels was determined according to the spectrophotometric method (*27*). Results of TEAC and ORAC were expressed as Trolox in µmol/g, while ICC was expressed as Na_2_EDTA in µmol/g. An average of three measurements was used for all calculations.

#### Antihypertensive activity

The antihypertensive activity of active agents released from the gels was determined by measuring their inhibitory effects on the angiotensin-converting enzyme (ACE) (*28*). Briefly, 0.25 U/mL ACE prepared in 0.01 mol/L phosphate buffer saline (PBS) at pH=7.0 was mixed with green tea extract solutions taken from the release medium. The enzyme-sample mixture was incubated for 15 min at 37 °C and the enzymatic reaction was initiated by adding 150 μL of 1.75 mmol/L FAPGG substrate solution. The assay was performed in 96-well microtiter plates (UV flat bottom, 8404; Thermo Fisher Scientific) and the absorbance was monitored at 340 nm for 30 min at 37 °C using microplate reader (Varioskan Flash; Thermo Fisher Scientific). ACE inhibition (%) was expressed in µg of captopril per g of gel. An average of three measurements was used for all calculations.

#### Antidiabetic activity

The antidiabetic activity of active agents released from the gels against human salivary α-amylase (HSA) and α-glucosidase (AGH) enzymes was determined according to Koh *et al.* (*29*). Both HSA and AGH inhibitions were calculated according to the following equation:

Inhibition*=*{[(*A*_control_*-A*_control blank_)-(*A*_sample_*-A*_sample blank_)]/(*A*_control_*-A*_control blank_)}*∙*100 */3/*

where *A*_control_, *A*_control blank_, *A*_sample_ and *A*_sample blank_ are the absorbances measured for the reaction mixtures prepared with the buffer using the active enzyme, with the buffer using the inactive enzyme, with the sample using the active enzyme, and with the sample using the inactive enzyme, respectively. An average of three measurements was used for the calculations. Results were expressed in µmol acarbose equivalent per g of gel.

#### Antiproliferative properties

The Caco-2 cells were cultured in minimum essential medium (MEM: Sigma-Aldrich, Merck, St. Louis, MO, USA) containing 15% foetal bovine serum (FBS; Gibco, Thermo Fisher Scientific, São Paulo, Brazil), 100 U/mL penicillin-streptomycin solution (Gibco, Thermo Fisher Scientific, Waltham), 1% non-essential amino acids (Gibco, Thermo Fisher Scientific, New York, NY, USA) and 1% sodium pyruvate (Gibco, Thermo Fisher Scientific, New York) in a humidified incubator with 5% CO_2_ at 37 °C. The Caco-2 cells (5000 cells/well) were plated into 96-well plates and allowed to grow for 24 h. Then, different mass (0, 21, 62, 123.6 or 247.2 µg) of samples was added into the wells that contained 0.2 mL of MEM (0, 105, 310, 618 or 1236 µg sample per mL of MEM). The wells were then incubated for 48 h at 5% CO_2_ and 37 °C. The cell toxicity was determined by CCK-8 (Sigma-Aldrich, Merck) assay according to the manufacturer’s instructions. Colour changes were measured at 450 nm, using a Varioscan plate reader (Multiscan Go; Thermo Fisher Scientific, Waltham). The absorbance from non-treated Caco-2 cells (control group) was taken as 100% viable and the viability of sample-treated cells was calculated as the percentage of the control group. An average of three measurements was used for the calculations.

### Statistical analysis

One-way analysis of variance (ANOVA) was performed to process the data of gel samples using Minitab Statistical Software for Windows, v. 17 (*30*). The normal distribution of samples was checked using the Shapiro-Wilk test. For the data that do not fit the normal distribution, the Kruskal-Wallis test was conducted using IBM SPSS Statistics for Windows, v. 23.0 (*31*). Statistical differences among the mean values were compared with multiple range test at a significance level of p<0.05.

## RESULTS AND DISCUSSION

### Effect of composite gel making on mechanical properties of gelatine gels

**Fig. S1:**
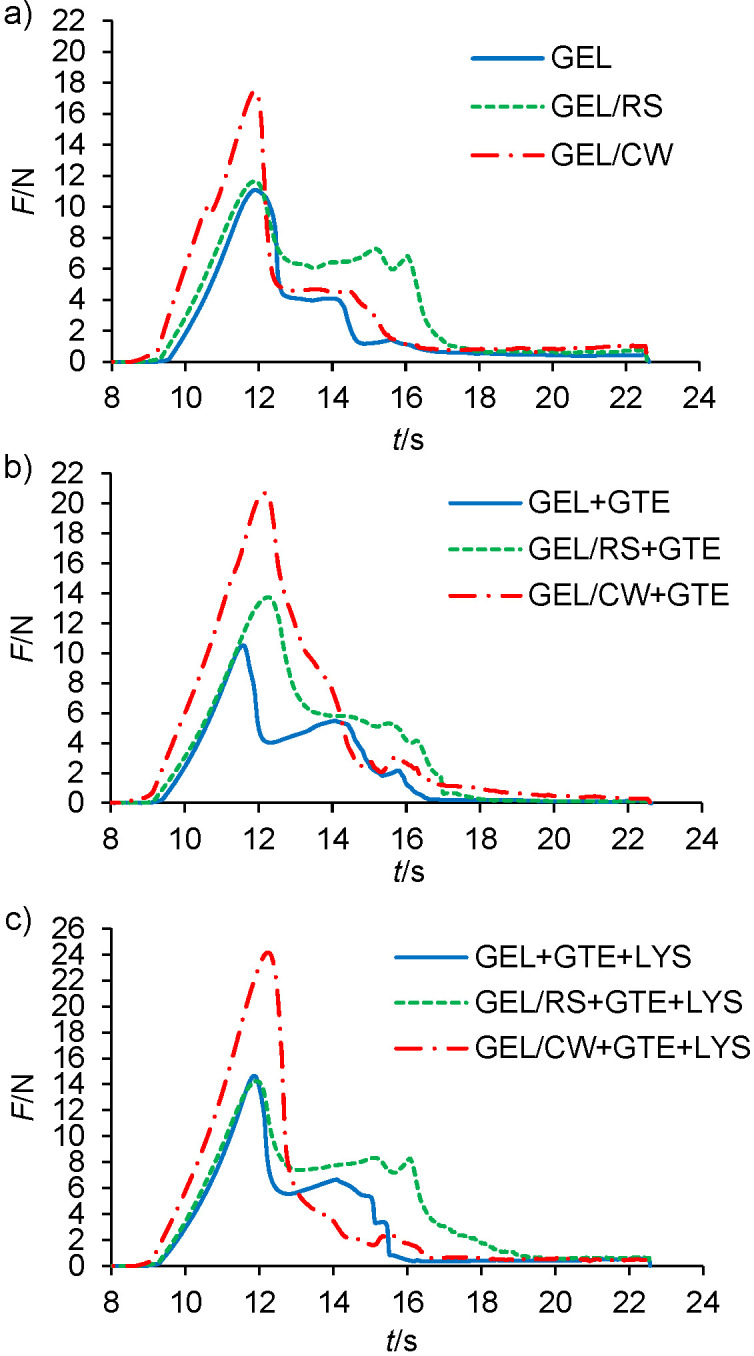
Force *vs* time graphs of: a) control gels, b) gels with green tea extract, and c) gels with lysozyme and green tea extract combination during shear test. GEL=gelatine, RS=rice starch, CW=candelilla wax, LYS=lysozyme, GTE=green tea extract

The mechanical properties of different gels were characterised by shear test and the calculated parameters are given in Table 1. The force *vs* time graphs of each gel are given in Fig. S1. The gelatine/wax gels showed significantly higher firmness, work to shear, and cutting strength than corresponding gelatine gels (p<0.05). No significant differences were determined between the work to shear of control gelatine/wax and gelatine/starch gels (p≥0.05), but all active gelatine/wax gels showed higher work to shear than corresponding active gelatine/starch gels. Moreover, all gelatine/wax gels (control or active) showed significantly higher firmness and cutting strength than gelatine/starch gels (p<0.05). The firmness and cutting strength of gelatine/starch and gelatine gels with green tea extract or lysozyme and green tea extract combination were similar, but all gelatine/starch gels showed higher work to shear than their corresponding gelatine gels. These results clearly showed that the most mechanically stable gel is gelatine/wax followed by gelatine/starch and gelatine gels. Also, the addition of green tea extract did not affect the work to shear of gelatine and gelatine/starch gels, but addition of lysozyme and green tea extract combination increased the work to shear of both gels. In contrast, the addition of green tea extract increased firmness, cutting strength, and work to shear of gelatine/wax gels significantly (p<0.05). These results clearly showed that the green tea extract increased the networking of the gelatine/wax gels. It seemed that the amphiphilic green tea phenolic fractions created some interactions both with gelatine and candelilla wax, and this supported the mechanical stability of the gelatine/wax gels. Different reports in the literature related to the affinity of green tea catechins to hydrophobic lipids support this hypothesis (*32*-*34*). The hydroxyl groups (-OH) of polyphenols could form extensive hydrogen bonding with carbonyl groups (C=O) of gelatine proteins (*35*), while at the same time their hydrophobic phenolic groups like aromatic rings (*e.g.* those of catechins like epigallocatechin gallate and epicatechin gallate) might create hydrophobic interactions with wax particles distributed within the gelatine gel matrix. Finally, in all gels the addition of green tea extract or lysozyme and green tea extract combination did not affect firmness and cutting strength. However, a significant increase in the work to shear was observed in all gels when lysozyme and green tea extract combination was added instead of green tea extract (p<0.05). The capacity of lysozyme to interact with gelatine (*1*) and polyphenols (*36*) has been well documented. However, overall results clearly show that the addition of lysozyme and green tea extract combination instead of green tea extract had no considerable effect on major mechanical properties of gelatine gel such as firmness and cutting strength.

**Table 1:** Shear test properties of gels

Gel sample	Firmness/N	Work to shear/(N∙s)	Cutting strength/(N/mm)
GEL	(10.9±0.6)^c^	(33.3±1.3)^e^	(0.44±0.02)^c^
GEL+GTE	(10.8±0.7)^c^	(31.6±1.2)^e^	(0.43±0.03)^c^
GEL+LYS+GTE	(14.4±0.5)^bc^	(41.94±1.05)^d^	(0.58±0.02)^bc^
GEL/RS	(13.1±1.2)^c^	(51.0±2.0)^c^	(0.52±0.05)^c^
GEL/RS+GTE	(13.8±0.7)^bc^	(49.4±1.4)^c^	(0.55±0.03)^bc^
GEL/RS+LYS+GTE	(14.3±0.7)^bc^	(57.98±1.4)^b^	(0.57±0.03)^bc^
GEL/CW	(17.5±1.6)^b^	(50.4±2.1)^c^	(0.71±0.06)^b^
GEL/CW+GTE	(22.8±2.7)^a^	(60.2±4.4)^b^	(0.9±0.1)^a^
GEL/CW+LYS+GTE	(24.9±1.7)^a^	(72.5±2.1)^a^	(0.99±0.07)^a^

Different superscripted lower-case letters in the same column indicate significant difference (p<0.05). Values in the parentheses are presented as mean value±S.E. (*N*=8). GEL=gelatine, RS=rice starch, CW=candelilla wax, LYS=lysozyme, GTE=green tea extract

### Water-binding properties of different gels

**Fig. 1:**
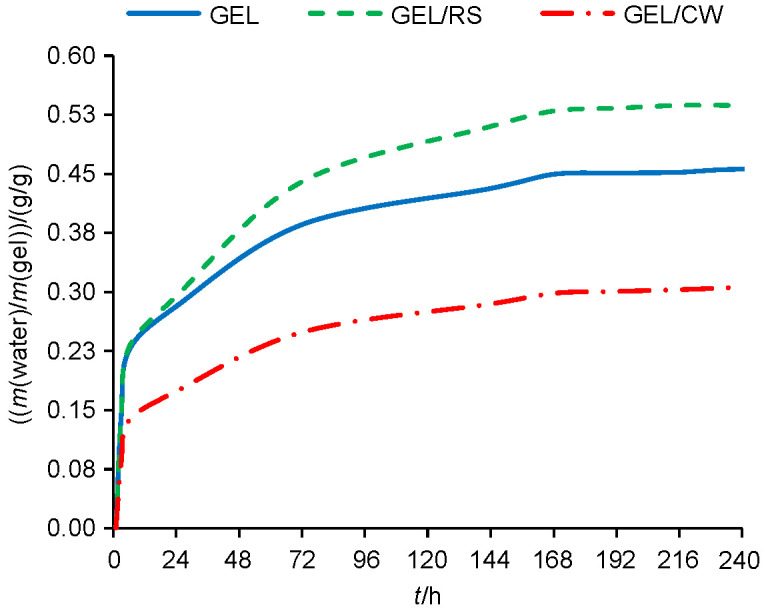
Water-binding capacities of the gels. GEL=gelatine, RS=rice starch, CW=candelilla wax

The swelling characteristics of different gels indicated that the addition of hydrophilic rice starch into gels increased their swelling rates, while composite with hydrophobic candelilla wax reduced the swelling rates (Fig. 1 and Table S1). Thus, at the equilibrium, the highest water-binding capacity (WBC) was determined for gelatine/starch gel (0.54 g/g), followed by gelatine (0.45 g/g) and gelatine/wax gel (0.31 g/g). These results clearly showed that the composite gels provide not only alternative mechanical properties but also considerably different WBCs that could be exploited to obtain gels with different textural properties.

### Effects of starch, wax and green tea extract on the colour of different gels

**Fig. S2:**
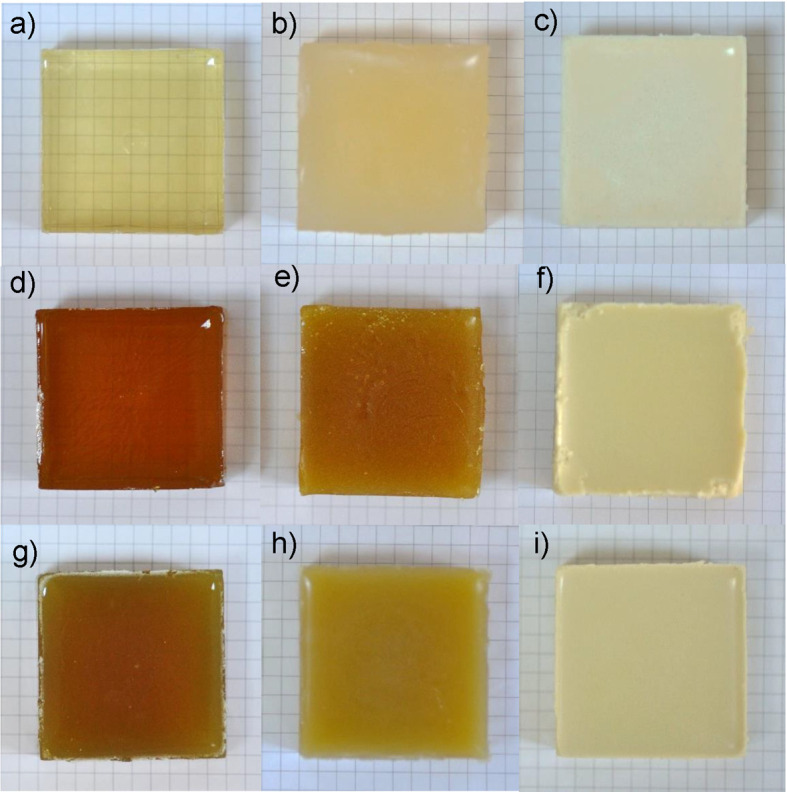
Photographs of the gels: a) gelatine, b) gelatine/starch, c) gelatine/wax, d) gelatine with green tea extract, e) gelatine/starch with green tea extract, f) gelatine/wax with green tea extract, g) gelatine with lysozyme and green tea extract combination, h) gelatine/starch with lysozyme and green tea extract combination, and i) gelatine/wax with lysozyme and green tea extract combination

**Fig. S3:**
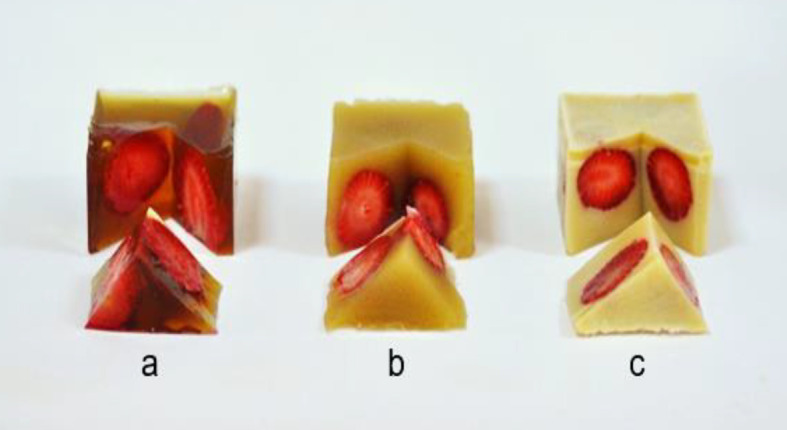
Photographs of gels with strawberries: a) gelatine gel with green tea extract, b) gelatine/starch gel with green tea extract, and c) gelatine/wax gel with green tea extract

The photos of the developed gels and their L*, a* and b* values determined by a digital colorimeter are presented in Fig. S2 and Table 2, respectively. The control gelatine gel is transparent and very light yellow (Fig. S2a), while control gelatine/starch gel is light yellow and non-transparent (Fig. S2b), and control gelatine/wax gel is non-transparent and white (Fig. S2c). The addition of green tea extract made the gelatine gels quite turbid and dark brownish (Fig. S2d) and the colour of gelatine/starch gels light brownish to greenish (Fig. S2e). Discolouration was also reported by Jamróz *et al.* (*37*), who added tea extracts into furcellaran-gelatin films. In contrast, green tea extract made gelatine/wax gels light yellow and successfully masked the brown colour formation (Fig. S2f). The same results were also observed in the photos of gels loaded with green tea extract when they were tested to obtain fruit jellies using strawberries as a model (Fig. S3). However, the addition of lysozyme and green tea extract combination gave less dark gelatine and gelatine/starch gels than the same gels with green tea extract (Fig. S2g and Fig. S2h). A reduction was also observed in yellowness of gelatine/wax gel by use of lysozyme and green tea extract combination instead of green tea extract (Fig. S2i). Thus, it appears that the lysozyme showed some protective effect on oxidation of polyphenols possibly due to its amino acid side chains having antioxidative properties (*38*). The darkening observed in the gels with the addition of green tea extract correlated inversely with their L* values. The highest L* values were determined in gelatine/wax control gel. The addition of green tea extract or lysozyme and green tea extract combination reduced the L* values of gelatine/wax gels, but these gels still showed 2- to 4-fold higher L* than the gelatine and gelatine/starch gels with green tea extract, and lysozyme and green tea extract combination. Also, it should be noted that L* values of all gels with lysozyme and green tea extract combination were significantly higher than those of gels with green tea extract. This result showed parallelism with the appearances of gels in photos that suggested a potential protective effect of lysozyme on green tea polyphenols. The overall results suggested that the numerous tiny rice starch and candelilla wax particles within composite gels prevented the passing of light from the gels, and this masked the colour originating from the green tea extract in the gels. It also appeared that the limited light contact of green tea polyphenols in composite gels also prevented their darkening with photodegradation in the presence of gelatine reactive groups (*39*). A considerable redness (a*) was observed only in the gelatine gels with green tea extract, while other gels were close to neutral or they showed a slightly greenish colour. The yellowness (b*) of composite gels increased significantly by the addition of green tea extract, while gelatine gels with green tea extract, and with lysozyme and green tea extract combination showed slight and moderate reductions in their yellowness, respectively.

**Table 2:** Colour properties of gels

Gel sample	L*	a*	b*
GEL	(37.5±0.2)^d^	(0.62±0.04)^b^	(14.8±0.2)^c^
GEL+GTE	(19.9±0.3)^h^	(6.3±0.3)^a^	(13.1±0.5)^d^
GEL+LYS+GTE	(25.04±0.08)^f^	(-0.99±0.06)^e^	(7.9±0.2)^f^
GEL/RS	(25.7±0.2)^f^	(-0.02±0.01)^c^	(2.10±0.04)^g^
GEL/RS+GTE	(24.2±0.32)^g^	(-0.44±0.03)^d^	(7.6±0.3)^f^
GEL/RS+LYS+GTE	(31.71±0.05)^e^	(-1.66±0.007)^f^	(11.1±0.1)^e^
GEL/CW	(89.4±0.2)^a^	(-0.35±0.02)^d^	(13.29±0.07)^d^
GEL/CW+GTE	(77.2±0.3)^c^	(-0.74±0.05)^e^	(31.9±0.1)^a^
GEL/CW+LYS+GTE	(79.8±0.3)^b^	(-0.98±0.03)^e^	(25.1±0.5)^b^

Different superscripted lower-case letters in the same column indicate significant difference (p<0.05). Values in the parentheses are presented as mean value±S.E. (*N*=5). GEL=gelatine, RS=rice starch, CW=candelilla wax, LYS=lysozyme, GTE=green tea extract

### Release profiles of lysozyme and green tea extract from different gels

**Fig. 2:**
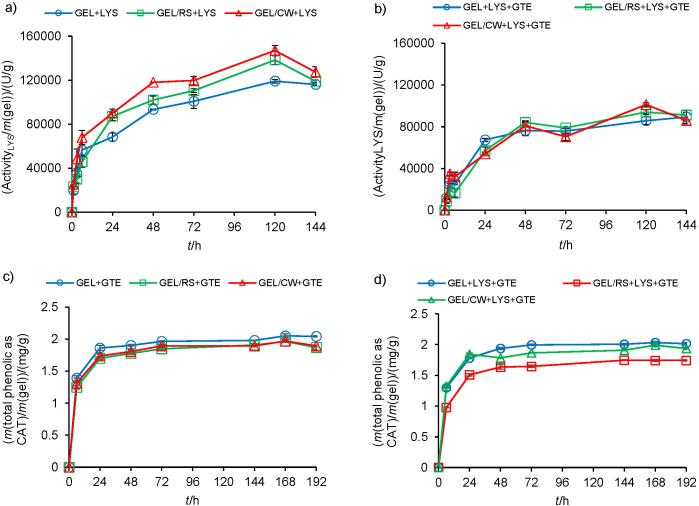
Release profiles of: a) lysozyme released from gels with lysozyme, b) lysozyme released from gels with lysozyme and green tea extract combination, c) phenolic compounds released from gels with green tea extract, and d) phenolic compounds released from gels with lysozyme and green tea extract combination. GEL=gelatine, RS=rice starch, CW=candelilla wax, LYS=lysozyme, GTE=green tea extract, CAT=catechin

The release profiles and release parameters of lysozyme and green tea extract from different gels are presented in Fig. 2 and Table 3, respectively. The initial activity of lysozyme and initial green tea phenolic content of gels achieved by loading 1% of both active compound preparations were  744 194 U and 3.29 mg catechin equivalents per g of gels, respectively. The gels incorporated with lysozyme showed similar release profiles (Fig. 2a). However, the recovery of lysozyme from gelatine, gelatine/starch, and gelatine/wax gels changed between 16 and 20%. This finding indicated that at the pH of gels (~6.0), the gelatine formed negative charges (pI=5.0-7.5) that bound a significant amount of positively charged lysozyme (pI=11.4) by charge-charge interactions. The total activities of released lysozyme from gelatine/starch and gelatine/wax gels were similar to each other, and they were 16 and 23% higher than that from gelatine gel, respectively. The higher total released lysozyme activities of composite gels might be favoured by the altered structure of the gelatine gel matrix by rice starch granules and candelilla wax particles. It seemed that the rice starch and candelilla wax incorporated into gels created weaker interaction with lysozyme than gelatine. Thus, this increased the fraction of soluble lysozyme within the gels. In contrast, in the presence of green tea extract, the gels again showed similar lysozyme release profiles, but the lysozyme recoveries of different gels dropped between 12 and 14% (Fig. 2b). Thus, it is evident that the combination of lysozyme with green tea extract increased the binding of lysozyme onto the gelatine gel matrix. The -OH groups of phenolic compounds are capable of forming extensive H-bonds with carbonyl groups of proteins (*40*). Therefore, it seemed that the polyphenols in green tea extract caused the formation of crosslinked bonds between the lysozyme and green tea extract. Moreover, increased crosslinking within gelatine gel matrix might also have caused physical trapping of lysozyme within the film matrix. The initial lysozyme release rates of gelatine, gelatine/starch and gelatine/wax gels without green tea extract were 1.4-, 1.2- and 1.8-fold higher than similar gels containing lysozyme and green tea extract combination, respectively. This finding supported the hypothesis that gel networking increased due to interaction of green tea extract polyphenols with gelatine. This hypothesis complies well with previous reports of Arcan and Yemenicioğlu (*41*) and Zhu *et al.* (*42*), who showed that the phenolic crosslinking is effective to sustain lysozyme release from zein and gelatine films, respectively.

**Table 3:** Lysozyme and green tea extract release properties of different gels in distilled water at 4 °C

Gel sample	Max. released activity/(U/g)^1^	Initial release rates/(U/(g∙h))^2^	Recovery/%^4^
GEL+LYS	(119071±1561)^b^	10055	16.0
GEL+LYS+GTE	(89283±6204)^d^	7099	12.0
GEL/RS+LYS	(138314±4016)^a^	8078	18.6
GEL/RS+LYS+GTE	(94130±2222)^cd^	6624	12.6
GEL/CW+LYS	(147070±4500)^a^	12568	19.7
GEL/CW+LYS+GTE	(101241±1766)^c^	6746	13.6
	Max. phenolics released expressed as catechin/(mg/g)^1^	Initial release rates expressed as catechin /(mg/(g·h))^3^	Recovery/%^4^
GEL+GTE	(2.05±0.06)^a^	0.087	62.2
GEL+LYS+GTE	(2.03±0.04)^a^	0.083	62.6
GEL/RS+GTE	(1.96±0.02)^a^	0.079	59.5
GEL/RS+LYS+GTE	(1.74±0.03)^b^	0.069	52.8
GEL/CW+GTE	(1.97±0.05)^a^	0.081	59.8
GEL/CW+LYS+GTE	(1.99±0.02)^a^	0.085	60.4

Different lower-case letters in superscript in the same column indicate significant difference (p<0.05). Values in the parentheses are presented as mean value±S.E. (*N*=3). ^1^Maximum released lysozyme activity and phenolic content in distilled water were reached at the 120th hour and on the 15th day, respectively. ^2^Time periods (h) of data used in the best fit were between 0 and 6 h (R^2^=0.87-0.99). ^3^Time periods (h) of data used in the best fit were between 0 and 24 h (R^2^=0.56-0.71). ^4^The addition of 1% lysozyme and green tea extract to the gels provided 744 194 U initial lysozyme activity and 3.29 mg catechin equivalent initial total polyphenols per g of gels. GEL=gelatine, RS=rice starch, CW=candelilla wax, LYS=lysozyme, GTE=green tea extract

The phenolic release profiles of gels are presented in Fig. 2c and Fig. 2d. The release tests clearly showed that gelatine, gelatine/starch and gelatine/wax gels showed similar green tea polyphenol release profiles expressed as catechin, with initial release rates from 0.069 to 0.087 mg/(g∙h). It was also important to note that the release rates of green tea extract were much faster than those of lysozyme and reached an equilibrium almost within 24 h. The total amounts of green tea polyphenols released from different gels were also similar and their phenolic recovery corresponded to almost 53-63% of total green tea polyphenols added into the gels. These results showed that the amount of free green tea extract in the gels and green tea extract release rates of the gels were not affected by the presence of candelilla wax and rice starch when the gels swelled in distilled water. To better understand the significance of phenolic content released by the gels, the results were also expressed by considering the phenolic content of one serving portion of green tea. According to Arcan and Yemenicioğlu (*43*), who applied similar testing methods, the one serving portion (200 mL) of green tea contains 140 mg gallic acid (or 61.7 mg catechin) equivalent of polyphenols. Thus, the total phenolic content released from 30 to 35 g portion of green tea extract-loaded gelatine, gelatine/starch and gelatine/wax gels is equivalent to that in one serving portion of green tea.

### Antimicrobial activity of different gels against L. innocua

The results of antimicrobial tests conducted at 4 °C for 48 h with gelatine, gelatine/starch and gelatine/wax gels with lysozyme, green tea extract or lysozyme and green tea extract combination in broth media against *L. innocua* are presented in Table 4. A 1.5-1.9 log increase in the *Listeria* load was observed in the control culture and the control gels lacking any active agents within 48 h. Gelatine and gelatine/starch gels with green tea extract were unable to inhibit *Listeria* growth, thus, this caused a significant (0.8-0.9 log) increase in microbial load within 48 h (p<0.05). In contrast, gelatine/wax with green tea extract showed antimicrobial effect and prevented significant increase of *Listeria* load during the 48 h of incubation (p<0.05). The antimicrobial effect of green tea extract against *L. monocytogenes* has been demonstrated before (*44*-*46*). Thus, the lack of significant antimicrobial effect of green tea extract released from gelatine and gelatine/starch gels could be related to its modifications (*e.g*. oxidation and polymerisation condensation) or interactions within these gels. In contrast, it appeared that the antimicrobial phenolic green tea extract fractions dispersed or solubilised within the hydrophobic wax particles in gelatine/wax gels underwent very limited modifications and/or interactions that diminished their antimicrobial properties. It was also found that all gels with lysozyme and lysozyme and green tea extract combination inhibited the growth of *Listeria* within 48 h. No significant differences were found between the antilisterial properties of similar gels with lysozyme and lysozyme and green tea extract combination (p≥0.05). Thus, it seemed that the reduced lysozyme release in the presence of green tea extract prevented the detection of any additive or synergetic antilisterial effects between these two active compounds within the gels. However, it is important to note that gelatine/starch gels with lysozyme and green tea extract combination, and gelatine/wax with lysozyme were the only gels that caused a significant reduction (approx. 0.5 log) in initial *Listeria* loads within 48 h.

**Table 4:** The antimicrobial activity of the gels against *Listeria innocua* during 48 h of storage at 4 °C

Gel sample	*N*/(log CFU/mL)*					
		*t*/h			
0		24		48	
Control		(3.8±0.2)^a,B^		(4.2±0.2)^a,B^		(5.6±0.5)^a,A^	
GEL		(3.2±0.6)^a,B^		(3.8±0.2)^bcde,B^		(4.8±0.4)^bc,A^	
GEL+LYS		(3.2±0.6)^a,A^		(3.52±0.4)^ef,A^		(3.3±0.2)^fg,A^	
GEL+GTE		(3.65±0.06)^a,B^		(3.9±0.38)^bcd,B^		(4.6±0.2)^cd,A^	
GEL+LYS+GTE		(3.4±0.1)^a,A^		(3.6±0.1)^cdef,A^		(3.4±0.1)^f,A^	
GEL/RS		(3.6±0.2)^a,C^		(4.1±0.1)^ab,B^		(5.06±0.03)^b,A^	
GEL/RS+LYS		(3.3±0.4)^a,A^		(3.4±0.3)^f,A^		(3.02±0.08)^g,A^	
GEL/RS+GTE		(3.0±0.7)^a,B^		(4.0±0.2)^abc,A^		(3.8±0.2)^e,A^	
GEL/RS+LYS+GTE		(3.5±0.2)^a,A^		(3.6±0.2)^def,A^		(3.05±0.07)^g,B^	
GEL/CW		(3.0±0.7)^a,B^		(4.1±0.1)^ab,A^		(4.4±0.2)^d,A^	
GEL/CW+LYS		(3.6±0.2)^a,A^		(3.58±0.08)^def,A^		(3.05±0.08)^g,B^	
GEL/CW+GTE		(3.4±0.4)^a,A^		(3.9±0.4)^bcd,A^		(3.03±0.05)^g,A^	
GEL/CW+LYS+GTE		(3.0±0.6)^a,B^		(3.8±0.2)^bcdef,A^		(3.28±0.03)^fg,AB^	

Different lower-case letters in superscript in the same column indicate significant difference (p<0.05). Different superscripted capital letters in the same row indicate significant difference (p<0.05). *Values in the parentheses are presented as mean value±S.E. (*N*=3). GEL=gelatine, RS=rice starch, CW=candelilla wax, LYS=lysozyme, GTE=green tea extract

### Bioactive properties of gelatine and its composite gels

#### ORAC, TEAC and ICC activities of different gels

**Fig. 3:**
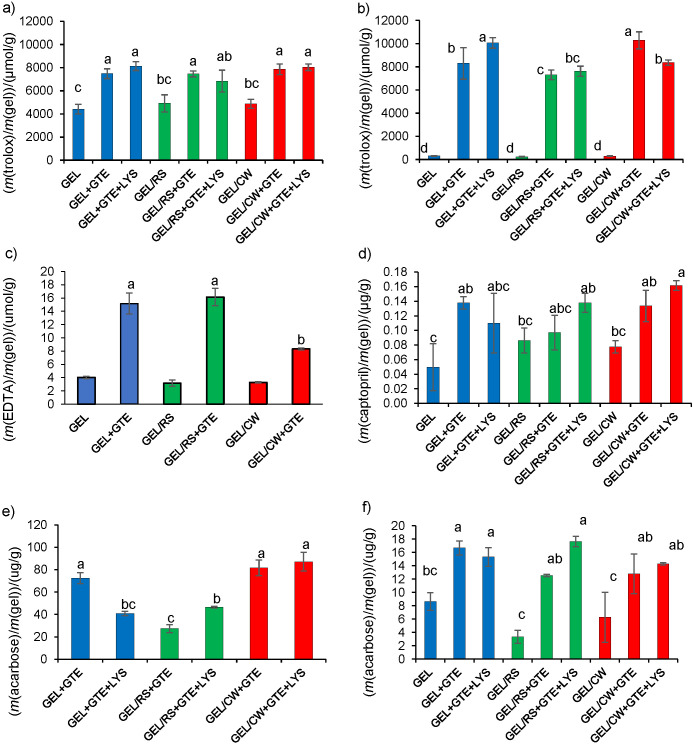
Antioxidant activities: a) oxygen radical absorbance capacity (ORAC), b) Trolox equivalent antioxidant capacity (TEAC) and c) the iron-chelating capacity (ICC), and d) antihypertensive, and e and f) antidiabetic (against HSA and AGH, respectively) activities of the gels. Different lower-case letters indicate significant difference (p<0.05). GEL=gelatine, RS=rice starch, CW=candelilla wax, LYS=lysozyme, GTE=green tea extract

The antioxidant properties of gels are presented in Figs. 3a-c. The control gels of gelatine, gelatine/starch and gelatine/wax lacking green tea extract showed considerable inherent ORAC values that were equivalent to 54 to 71% of those obtained for gels containing green tea extract or lysozyme and green tea extract combination (Fig. 3a). The inherent ORAC of gels could originate from soluble antioxidant protein and peptide residues released from the gelatine gel matrix (*47*). In fact, the water-soluble protein contents (assayed by the Bradford method) released from the gels incubated in distilled water at 4 °C changed between 99 and 119 µg/g. No considerable TEAC and ICC were measured for the control gels. The addition of green tea extract or lysozyme and green tea extract combination caused a 1.4- to 2-fold increase in ORAC of the gels, while the increase of TEAC of gels was 28- to 32-fold (Fig. 3b). The ORAC values of green tea extract containing gelatine, gelatine/starch and gelatine/wax gels were not significantly different (p*≥*0.05) regardless of the presence of lysozyme. In contrast, the TEAC of the gelatine gel with lysozyme and green tea extract combination and gelatine/wax gel with green tea extract were 20 to 41% higher than those of the other gels with green tea extract or lysozyme and green tea extract combination. The gels did not show such a great variation in their total soluble phenolic contents (Table 3). However, the green tea extract might contain eight different forms of catechins that vary in antioxidant potential even for epimer pairs (*48*). Therefore, the variations in TEACs of gels could be due to their compositional and morphological differences that affected the release profiles of phenolic compounds. Although the highest TEACs were determined for gelatine gels with lysozyme and green tea extract combination and gelatine/wax with green tea extract, green tea polyphenols in gelatine/wax gel did not show browning and had better antimicrobial activity than all other green tea extract-loaded gels (Table 4). It appeared that the gelatine/wax got a protective effect on functional groups of green tea polyphenols such as galloyl residues that are responsible for antimicrobial and antioxidant activity of catechins (*49*). Moreover, it seemed that the polyphenols solubilised or dispersed in wax particles are protected from undesired interactions and modifications, thus, they maintained not only their antioxidant and antimicrobial activity, but they also avoided reactions causing discolouration.

The ICC of lysozyme-containing gels could not be determined due to the turbidity formed in the reaction mixture caused by the lysozyme. However, measurements with gels containing green tea extract indicated that ICC of gels expressed as EDTA equivalents (ranged between 8.32 and 16.15 µmol/g) increased 2.5- to 5-fold with the addition of green tea extract (Fig. 3c). The overall results showed that green tea extract caused substantial increases in TEAC of the gels, while it caused limited improvements in ORAC and ICC of the gels.

#### Antihypertensive and antidiabetic activities of different gels

The results showed that the soluble proteins and peptides in control gelatine, gelatine/starch and gelatine/wax gels showed inherent antihypertensive activities that are not significantly different from each other (p*≥*0.05) (Fig. 3d). It seemed that the gelatine peptides, known for their antihypertensive activity, might have dissolved into release media and showed an ACE inhibitory effect (*50*, *51*). The addition of green tea extract to gels caused a limited increase in their antihypertensive activities except for gelatine with green tea extract and gelatine/wax with lysozyme and green tea extract combination. However, ACE inhibition determined as the IC_50_ of captopril was 0.019 µg/mL (0.089 µM). Thus, it is important to note that the captopril equivalents released from one g of gelatine, gelatine/starch or gelatine/wax gel loaded with green tea extract or lysozyme and green tea extract combination were 5- to 8.4-fold higher than IC_50_ of captopril.

Due to the soluble gelatine protein fractions in gels, control gelatine, gelatine/starch and gelatine/wax gels showed similar inherent HSA inhibition capacities (p*≥*0.05) (Fig. 3e). In contrast, the control gels did not show any inhibitory effect on α-glucosidase (AGH) (Fig. 3f). The addition of green tea extract or lysozyme and green tea extract combination into gels caused significant increases in AGH- and HSA-based antidiabetic activities of all gels. The HSA-based antidiabetic activities of gels loaded with green tea extract and lysozyme and green tea extract combination did not show significant variations. In contrast, the gelatine gels with green tea extract, and gelatine/wax both with green tea extract or lysozyme and green tea extract combination showed significantly higher AGH-based antihypertensive activities than other gels. This result once more suggested differences among bioactive profiles of phenolics released from different gels. Moreover, the acarbose equivalents released from one g of gels loaded with green tea extract or lysozyme and green tea extract combination were 6- to 8-fold and 5- to 11-fold higher than IC_50_ of acarbose determined for HSA (IC_50_=2.1 µg/mL or 3.24 µM) and AGH (IC_50_=8.07 µg/mL or 12.50 µM), respectively.

#### Antiproliferative activity of different gels on Caco-2 cells

**Fig. 4:**
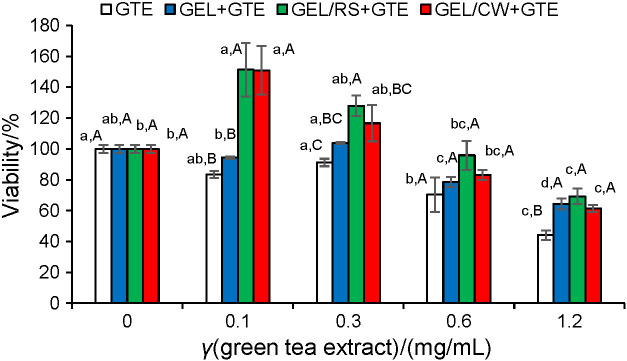
Effect of green tea extract and lyophilized release media of gels with green tea extract on viability of Caco-2 cells. Different lower-case letters indicate significant difference between the viability values of a single extract at different concentrations (p<0.05). Different superscripted capital letters indicate significant differences between the viability values of different extracts at the same concentration (p<0.05). GEL=gelatine, RS=rice starch, CW=candelilla wax, LYS=lysozyme, GTE=green tea extract

The antiproliferative activities of pure green tea extract and lyophilized release test media from gelatine, gelatine/starch, and gelatine/wax gels containing green tea extract were tested on Caco-2 cells and the results are given in Fig. 4. Different rates of cellular proliferative activities were observed for lyophilized release media of green tea extract-loaded gelatine/starch and gelatine/wax gels at 0.1 and 0.3 mg/mL. However, the lyophilized release medium of gelatine gels with green tea extract did not show a significant proliferative effect on Caco-2 cell growth. On the other hand, when green tea extract or lyophilized release medium concentration (≥0.6 mg/mL) was increased, the growth rate of Caco-2 cells was significantly reduced. The pure green tea extract was used as a control of cell culture experiments and its IC_50_ was determined at the level of 1.12 mg/mL. The lyophilized release media from gelatine and gelatine/wax gels containing green tea extract showed similar antiproliferative activities with IC_50_ values at 1.67 and 1.65 mg/mL, respectively. The medium from gelatine/starch gel containing green tea extract (IC_50_=2.37 mg/mL) showed the lowest antiproliferative activity. Thus, it is important to note that 1.2 to 1.5 g of gelatine, gelatine/starch, and gelatine/wax gels could release green tea extract phenolics equivalent to IC_50_ values determined for Caco-2 cells. These results clearly indicated that the gels with green tea extract added have the ability to reduce Caco-2 cell growth.

## CONCLUSIONS

The use of composites of gelatine with candelilla wax caused dramatic increases in the mechanical stability of the obtained gels, and these gels effectively prevented the darkening of loaded green tea polyphenols. Moreover, composite gels formed with candelilla wax showed protective effects on antimicrobial, antioxidant and antidiabetic activities of green tea polyphenols. The gelatine composites with rice starch had less pronounced positive benefits on colour and mechanical properties of gels than those obtained with candelilla wax, but these gels caused a considerable increase in water-binding properties of gelatine gels. Composite gel made with rice starch also had some positive effects on the antimicrobial performance of the gels, but this caused no improvements in the bioactive properties of the gels. This work provided basic data for tailoring of physical properties, release profile and active properties of gels loaded with bioactive compounds. The bioactive gels could be employed for developing alternative functional gel-based foods and edible active filling, glazing, coating or pad materials.

## Figures and Tables

**Table S1:** Water-binding capacity (WBC) of gels

Gel sample	WBC/(g/g)*
GEL	(0.453±0.003)^b^
GEL/RS	(0.54±0.01)^a^
GEL/CW	(0.310±0.005)^c^

*Values are presented as mean value±S.E. (*N*=3). Data refer to 10 days after water immersion of gels. Different superscripted lower-case letters indicate significant differences (p<0.05). GEL=gelatine, RS=rice starch, CW=candelilla wax
